# P56 Arresting a self-perpetuating crisis of AMR: onsite purification and decontamination of hospital waste and wastewater using the Pharmafilter system

**DOI:** 10.1093/jacamr/dlaf118.063

**Published:** 2025-07-14

**Authors:** P Kelly, E Gleeson

**Affiliations:** Pharmafilter Ltd UK & Ireland; Pharmafilter Ltd UK & Ireland

## Abstract

**Background:**

Antibiotics and resistant microbes enter the environment via hospital waste and wastewater, they evolve and spread, and eventually return to healthcare settings in more dangerous, less treatable forms. This is placing added pressure on already burdened hospitals by straining systems, treatment protocols, and endangering patients and staff alike. Pharmaceutical waste from humans often pass unchecked into our environment, creating a dangerous cycle of contamination that is damaging to the environment and is a significant public health threat. Every day, hospitals discharge vast quantities of wastewater loaded with antibiotics, active pharmaceutical ingredients, and metabolized drug residues. These compounds are specifically designed to act on biological systems, and even trace concentrations present a serious risk when they re-enter the environment. Traditional utility and wastewater treatment plants are not equipped to effectively remove these complex chemical compounds, and as a result, hospitals are unintentionally releasing micropollutants into public infrastructure, where they remain biologically active and contribute directly to the rise of antimicrobial resistance (AMR). The issue goes beyond just wastewater. Hospitals generate between 1200 and 2000 kilograms of solid waste per bed annually, including hazardous or infectious materials. Despite this, only 15 to 20 per cent of hospital waste is recycled. There is a necessity for onsite waste and wastewater management. The Pharmafilter system is designed to address this critical unmet need.

**Methods:**

The purpose of this study was to review the evidence for onsite waste and wastewater management within hospitals. To determine the key evidence a single point of failure approach to environmental AMR spread was considered which focused on two key areas: 1. On site removal of chemical and pharmaceuticals 2. On site elimination of biological micro-pollutants including pathogens and antibiotic resistance genes (ARGs).

**Results:**

On-site treatment is substantially more efficient in reducing antibiotic and ARG concentrations than municipal wastewater treatment. The Pharmafilter system allows for the removal of active pharmaceutical compounds below the detectable limit, and the complete removal of antibiotic-resistant bacteria and genes. The system offers unrivalled hospital generated effluent and wastewater biological purification.

**Conclusions:**

This situation of AMR environmental spread is only likely to worsen with rapid growth of the healthcare due to advancements in medicine, ageing populations and the increasing prevalence of chronic diseases. Hospitals need to rethink how we manage waste and wastewater —not just as a logistical issue but as a critical priority for public health. By treating wastewater onsite, a hospital can arrest a cycle of contamination by preventing the release of pharmaceutical ingredients, antibiotics (antimicrobials), micropollutants, organic and inorganic compounds, and harmful pathogens before they enter the environment. By reducing the manual transport of solid waste and integrating a proximity-based disposal system for these materials throughout the hospital, along with using single-use bedpans and urinals, there is an opportunity to address the impact of HAI on wards by reducing cross-contamination and hand contact moments.

